# Prognostic Role of p53 Immunohistochemical Status in Invasive Breast Cancer. A Retrospective Review of 1387 Cases With Luminal-Like/Her2 Negative Breast Tumors

**DOI:** 10.1093/oncolo/oyad309

**Published:** 2023-11-23

**Authors:** Gerti Dajti, Margherita Serra, Giovanni Cisternino, Claudio Ceccarelli, Alice Pellegrini, Marica Melina, Antonio De Leo, Donatella Santini, Mario Taffurelli, Simone Zanotti

**Affiliations:** Breast and General Surgery Unit, IRCCS Azienda Ospedaliero-Universitaria di Bologna, Bologna, Italy; Breast and General Surgery Unit, IRCCS Azienda Ospedaliero-Universitaria di Bologna, Bologna, Italy; Surgical Pathology Unit, IRCCS Ospedale San Raffaele di Milano, Milan, Italy; Department of Experimental, Diagnostic and Specialty Medicine–DIMES, Alma Mater Studiorum University of Bologna, Bologna, Italy; Breast and General Surgery Unit, IRCCS Azienda Ospedaliero-Universitaria di Bologna, Bologna, Italy; Breast and General Surgery Unit, IRCCS Azienda Ospedaliero-Universitaria di Bologna, Bologna, Italy; Department of Experimental, Diagnostic and Specialty Medicine–DIMES, Alma Mater Studiorum University of Bologna, Bologna, Italy; Surgical Pathology Unit, IRCCS Azienda Ospedaliero-Universitaria di Bologna, Bologna, Italy; Breast and General Surgery Unit, IRCCS Azienda Ospedaliero-Universitaria di Bologna, Bologna, Italy; Breast and General Surgery Unit, IRCCS Azienda Ospedaliero-Universitaria di Bologna, Bologna, Italy

**Keywords:** breast cancer, p53, luminal-like, survival

## Abstract

**Purpose:**

The aim of this study was to evaluate the prognostic role of p53 immunohistochemical (IHC) expression in a large cohort of patients with hormone receptors (HR)-positive/Her2-negative primary invasive breast cancer.

**Methods:**

Retrospective review of consecutive cases treated at our Breast Unit between 2003 and 2013. Patients were divided into 3 subgroups based on p53 IHC expression: null (0%), low (0.1%-49%), and high (≥50%) p53 expression.

**Results:**

A total of 1387 patients were included in the study with a median follow-up of 86 months. After adjusting for age, size, node status, lymphovascular invasion, progesterone, and Ki67 expression, only null p53 immunophenotype was associated with worse disease-free survival (DFS) (OR 1.74, 95% IC, 1.11-2.71, *P* = .015) and distant recurrence-free survival (DRFS) (OR 1.73, 95% IC, 1.04-2.90, *P* = .036). Null p53 impacted significantly DFS and DRFS also in patients with early breast cancer.

**Conclusions:**

p53 IHC expression affects survival and, thus can be a valuable tool in the management of patients with HR-positive/Her2-negative breast cancer

## Teaser

This study evaluated the prognostic role of p53 immunohistochemical expression in a large cohort of patients with hormone receptor-positive/Her2-negative primary invasive breast cancer.

**Implications for Practice**

Luminal-like/Her2-negative breast cancer is associated generally with good outcome but may also include high-risk tumors for which systemic therapy might be beneficial in addition to endocrine therapy alone. Immunohistochemical p53 expression has been used as a surrogate for TP53 mutations. Based on our result in a large homogeneous cohort, the lack of p53 expression (null p53) is associated with worse disease- and distant recurrence-free survival, with its impact appearing to be stronger for early breast cancer (T1N0M0). Thus, IHC p53 status can be a useful tool in deciding the most appropriate treatment approach in patients with luminal-like/Her2 breast cancer.

## Introduction

Hormone receptors (HR)-positive/Her2-negative tumors are the most frequent subtype of breast cancer. Despite a widely accepted more favorable outcome, they also include a subgroup of high-risk tumors for which systemic therapy might be beneficial in addition to the endocrine therapy alone. Clinicopathological features, as well as newly genomic tests specifically designed and validated to this purpose, are used in clinical practice to identify such cases.^1-4^

The p53 protein is a transcriptional factor codified by the *TP53* gene and plays a key role in multiple cellular pathways, either with its nuclear transcriptional activity or through non-transcriptional cytoplasmatic processes. In response to stress conditions such as DNA damage, metabolic impairments, or oncogenic transformation, p53 can regulate the cell cycle arrest, senescence, DNA repair, and angiogenesis.^5-9^*TP53* mutations are the most frequent genetic alteration in human cancer and occur in 20%-30% of all primary invasive breast cancers. *TP53* mutations are observed in 10%-20% of luminal-like tumors, more frequently among luminal B-like tumors.^10,11^ Missense mutations involving the DNA-binding domain, nonsense, and frameshift mutations have been associated with poor survival, although the prognostic role of *TP53* mutations remains still not fully clarified.^12-15^ In most cases, mutant-*TP53* leads to a more stabilized p53 protein that tends to accumulate in the nucleus, thus immunohistochemical (IHC) determination of p53 expression has been used as a surrogate for the presence of *TP53* mutations.^16-18^ Consequently, different cutoffs have been proposed to distinguish abnormal p53 expression. 10% and 50% are the most frequently reported cutoffs but neither has yet been validated. P53 accumulation has been associated with poor survival in patients with luminal-like breast cancer, but due to controversial results, different methods and surrogates used, p53 expression has not yet been validated as a reliable prognostic factor.^19-27^

The aim of the study was to explore the impact of p53 IHC expression on long-term outcome in a large cohort of patients treated for HR-positive/Her2-negative breast.

## Materials and Methods

The present study is a retrospective review of 1387 cases with HR-positive/Her2-negative primary breast cancer treated at Breast Unit, Policlinico di Sant’Orsola, Bologna, Italy, between January 2003 and December 2013. The study was approved by the local ethics committee (21/2015/O/OssN). Patients with available clinicopathological and follow-up information were included. Collected variables consisted of the patients’ age, tumor size, axillary lymph node status, histologic grade and type, lymphovascular invasion (LVI), IHC measurements of estrogen and progesterone receptors, Ki67 and p53 expression. All immunohistochemical markers were evaluated by computer-assisted image analysis. Node status was considered positive (LN+) if at least one macrometastasis was observed. The new St. Gallen criteria were used to classify luminal-like tumors. Luminal B-like tumors included neoplasms with either PR expression lower than 20% or Ki67 higher than 25%.^28^ The expression of p53 was immunohistochemically measured using mouse monoclonal antibody to p53 (Clone DO7; Ventana) and based on the percentage of stained cells all cases were divided into 3 subgroups: null (0%), low (0.1%-49%) and high (50%-100%). Early breast cancer was defined as a tumor of 2 cm or less with negative node status and no distant metastases (T1N0M0). Follow-up was defined as time between surgery and last available follow-up and expressed as the median value. Overall recurrence rate was defined as the portion of patients developing either local or distant recurrences. The primary endpoint was to investigate the prognostic impact of p53 expression on DFS and DRFS. The secondary endpoint was to evaluate such association in patients with early breast cancer. Events for the disease-free survival analysis were defined as the first disease recurrence, either local or distant, for the primary and-point and distant recurrences for the secondary endpoint.

Statistical analysis was carried out using the SPSS software (version 20.0, IBM). For comparison among groups, chi-square and Mann-Whitney tests were used as appropriate. Kaplan-Meier survival analyses were conducted to compare survival among groups. The final multivariate Cox regression model was built from the set of variables that reached *P* < .10 at the univariate analysis by removing predictors based on the *P*-value in a stepwise manner. The threshold for statistical significance of 2-tailed tests was set at *P* < .05.

## Results

A total of 1387 cases were included in this study, of which 786 (57%) were luminal A-like and 601 (43%) were luminal B-like tumors. Invasive ductal carcinoma was the most common histological type (1144 patients, 82.4%). Luminal B-like tumors were associated with larger size, higher histologic grade and more frequent axillary lymph node involvement, presence of LVI, null p53, or high p53 expression. Null p53 and high p53 expression were observed in 59 (4.3%) and 93 (6.7%) of all cases, respectively. Postsurgical hormonal and chemotherapy (CHT) were prescribed in 92% and 40% of the cases, respectively. CHT was more likely to be administered in patients with luminal B-like tumors. Clinicopathological data are presented in Table 1.

**Table 1.** Clinicopathological features in all patients.

	All patients *n* = 1387	Luminal A*n* = 786	Luminal B*n* = 601	*P*-value
Mean age (range)	63.6 (27-96)	63.6 (27-96)	63.8 (32-95)	.785
Mean size (range) in mm	18.2 (0.4-140)	16.2 (1-140)	20.9 (0.4-80)	<.001
Histological type				.479
Ductal	1144 (82%)	649 (83%)	495 (82%)	
Lobular-ductal	25 (2%)	18 (2%)	7 (2%)	
Lobular	218 (16%)	119 (15%)	99 (16%)	
Lymph nodes status^*^				<.001
Negative	871 (67%)	551 (74%)	320 (57%)	
Positive	435 (33%)	196 (26%)	239 (43%)	
Stage^*^				<.001
I	781 (57%)	520 (67%)	261 (44%)	
II	373 (27%)	176 (23%)	197 (34%)	
III	185 (14%)	75 (9%)	110 (19%)	
IV	22 (2%)	3 (1%)	19 (3%)	
Histologic grade^*^				<.001
Low/intermediate	997 (72%)	658 (84%)	339 (57%)	
High	386 (28%)	128 (16%)	258 (43%)	
LVI^*^				<.001
Absent	909 (73%)	565 (78%)	344 (68%)	
Present	329 (27%)	163 (22%)	166 (32%)	
P53 IHC expression				.001
Low	1235 (89.0%)	748 (95.2%)	487 (81.0%)	
High	93 (6.7%)	23 (2.9%)	70 (11.6%)	
Null	59 (4.3%)	15 (1.9%)	44 (7.3%)	
Postoperative therapy				
Hormone therapy^*^	830 (92%)	459 (91%)	371 (93%)	.446
Chemotherapy^*^	352 (40%)	163 (33%)	189 (48%)	<.001
Overall recurrence				
Yes	270 (19.5%)	98 (12.5%)	172 (28.6%)	.001
No	1117 (80.5%)	688 (87.5%)	429 (71.4%)	
Local recurrence				.011
Yes	99 (7%)	44 (6%)	55 (9%)	
No	1288 (93%)	742 (94%)	546 (91%)	
Distant recurrence				<.001
Yes	201 (15%)	69 (9%)	132 (22%)	
No	1185 (85%)	716 (91%)	469 (78%)	

^*^Data not available for all patients.

Median follow-up was 86 months. A total of 270 patients developed either local or distant recurrences with an overall recurrence rate of 19.5%. The most frequent sites of distant metastases were the skeletal system (7%), liver (6%), lung (4%), distant lymph node stations (2%), and the central nervous system (1%). Median interval from the time of surgery to the recurrence was 44 months (56 and 35 months for luminal A- and B-like, respectively (*P* < .001).

**Figure 1.**
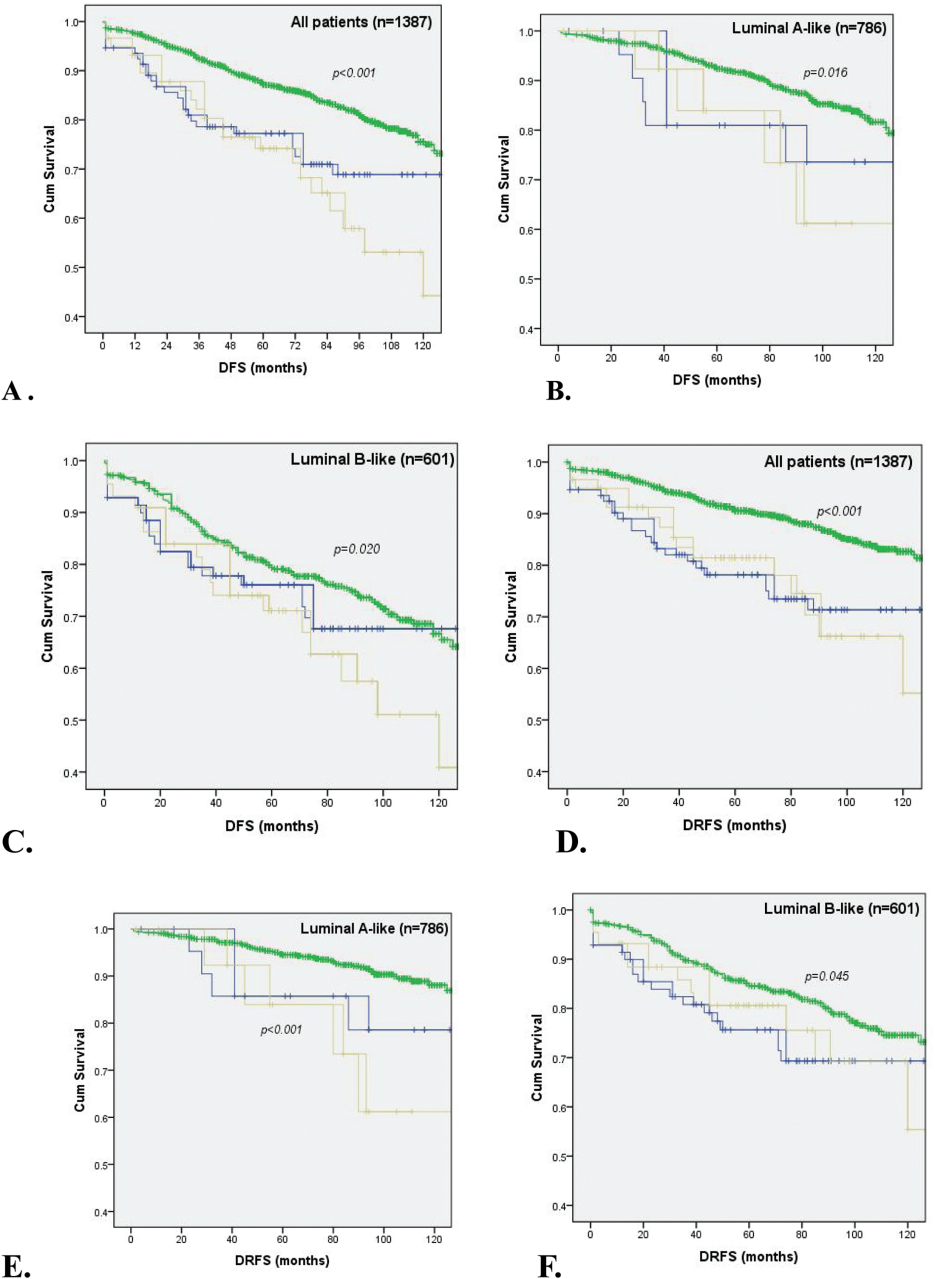
Kaplan-Meier survival curves according to p53 expression: low (green line **-----**), high (blue line **-----**), and null (yellow line **-----**) for DFS in all patients (**A**), luminal A- (**B**) and luminal B-like (**C**) and for DRFS in all patients (**D**), luminal A- (**E**) and luminal B-like (**F**).

Kaplan-Meier curves for DFS and DRFS are shown in Fig. 1. After adjusting for age, size, node status, histologic grade, LVI, PR and Ki67 expression, null p53 resulted an independent and significant factor associated with worse DFS and DRFS (OR 1.74, 95% IC, 1.11-2.71, *P* = .015 and OR 1.73, 95% IC, 1.04-2.90, *P* = .036, respectively). Univariate and multivariate Cox regression analyses are shown in Table 2.

**Table 2.** Cox proportional hazards models of DFS and DRFS in all patients.

Variables	Univariate analysis			Multivariate analysis		
	HR	95% IC	*P-*value	HR	95% IC	*P-*value
Disease-free survival						
Age	.990	.981-.999	.032	.987	.978-.997	.010
Size	1.029	1.025-1.034	<.001	1.025	1.019-1.030	<.001
LN+	1.061	1.051-1.072	<.001	1.043	1.031-1.056	<.001
Histologic grade	2.197	1.725-2.797	<.001	1.598	1.199-2.129	.001
LVI	1.532	1.154-2.030	.003	n. s.		
Ki67	2.769	2.168-3.536	<.001	1.697	1.263-2.281	<.001
PR	1.758	1.380-2.239	<.001	1.460	1.223-1.898	.005
Postoperative CHT	2.126	1.559-2.899	<.001	n. s.		
P53 IHC expression						
Low	—	—	—	—	—	—
High	1.699	1.136-2.542	*.010*	.918	.583-1.446	.713
Null	2.780	1.823-4.240	*.000*	1.735	1.111-2.710	.015
Distant recurrence-free survival						
Age	.984	.974-994	.003	.9844684	.974-.996	.006
Size	1.031	1.026-1.036	<.001	1.025992	1.020-1.032	<.001
LN+	1.064	1.054-1.075	<.001	1.04364	1.031-1.057	<.001
Histologic grade	2.426	1.834-3.209	<.001	1.603441	1.148-2.239	.006
LVI	1.498	1.075-2.087	.017	n. s.		
Ki67	3.304	2.496-4.374	<.001	1.854053	1.321-2.602	<.001
PR	1.922	1.453-2.542	<.001	1.593813	1.178-2.217	.003
Postoperative CHT	2.990	2.081-4.295	<.001	n. s.		
P53 IHC expression						
Low	—	—	—	—	—	—
High	2.204	1.441-3.371	<.001	1.047454	.641-1.71	.853
Null	2.927	1.795-4.771	<.001	1.732152	1.036-2.895	.036

Subsequently, we restricted our analysis to the group of patients with early breast cancer. We identified 732 cases with early breast cancer of which 489 (67%) were luminal A-like and 243 (33%) were B-like tumors. Clinicopathological data for patients with early breast cancer are shown in Table 3. Null p53 was significantly associated to a worse DFS and DRFS (OR 5.46, 95% IC, 2.52-13.24, *P* < .001 and OR 8.48, 95% IC, 2.87-25.01, *P* < .001 respectively). Univariate and multivariate regression analysis are shown in Table 4.

**Table 3.** Clinicopathological features of patients with early breast cancer.

	All patients *n* = 732	Luminal A *n* = 489	Luminal B *n* = 243	*P*-value
Mean age (range)	63.5 (27-88)	63.3 (27-88)	64.0 (37-88)	.521
Mean size in mm (range)	11.9 (0.4-20)	11.6 (1-20)	12.5 (0.4-20)	.007
Histological type				
Ductal	467 (64%)	405 (83%)	198 (81%)	
Ductal-lobular	17 (2%)	22 (4%)	6 (3%)	.600
Lobular	248 (34%)	62 (13%)	39 (16%)	
Histologic grade^*^				<.001
Low/intermediate	579 (79%)	424 (87%)	155 (64%)	
High	151 (21%)	65 (13%)	86 (36%)	
LVI^*^				.085
Absent	553 (81%)	382 (83%)	171 (77%)	
Present	126 (19%)	77 (17%)	49 (23%)	
P53 IHC expression				
Low	682 (93.2%)	472 (96.5%)	210 (86.4%)	
High	34 (4.6%)	11 (2.2%)	23 (9.5%)	<.001
Null	16 (2.2%)	6 (1.2%)	10 (4.1%)	
Postoperative therapy				
Hormone therapy^*^	426 (91%)	269 (90%)	157 (93%)	.239
Chemotherapy^*^	100 (22%)	54 (18%)	46 (28%)	.016
Overall recurrence				.325
Yes	59 (8%)	36 (7%)	23 (9%)	
No	673 (92%)	453 (93%)	220 (91%)	
Local recurrence				.919
Yes	37 (5%)	25 (5%)	12 (5%)	
No	695 (95%)	464 (95%)	231 (95%)	
Distant recurrence				.107
Yes	35 (5%)	19 (4%)	16 (7%)	
No	697 (95%)	470 (96%)	227 (93%)	

^*^Data not available for all patients.

**Table 4.** Cox proportional hazards models of DFS and DRFS in patients with early breast cancer.

Variables	Univariate analysis			Multivariate analysis		
	HR	95% IC	*P*-value	HR	95% IC	*P-*value
Disease-free survival						
Age	.986	.966-1.006	.157	n. s.		
Size	1.033	.981-1.088	.223	n. s.		
Histologic grade	3.121	1.880-5.183	<.001	2.401	1.392-4.143	.002
LVI	2.376	1.336-4.225	.003	1.849	1.020-3.349	.043
Ki67	1.037	1.016-1.060	.001	n. s.		
PR	.968	.547-1.711	.910	n. s..		
Postoperative CHT	1.864	.954-.3.641	.068	n. s.		
P53 expression						
Low	—	—	—	—	—	—
High	1.760	.694-4.461	.234	1.331	.465-3.810	.594
Null	7.878	3.357-18.491	<.001	5.459	2.252-13.236	<.001
Distant recurrence-free survival						
Age	.957	.950-1.001	.058	n. s.		
Size	1.088	1.015-1.166	.017	1.074	1.000-1.153	.049
Histologic grade	2.979	1.528-5.809	.001	2.164	1.065-4.392	.033
LVI	1.779	.792-3.993	.163	n. s.		
Ki67	1.044	1.017-1.071	.001	n. s.		
PR	1.499	.745-3.015	.256	n. s.		
Postoperative CHT	2.522	1.083-5.874	0.032	n. s.		
P53 expression						
Low	—	—	—	—	—	—
High	2.856	.991-8.233	.052	1.771	.585-5.363	.312
Null	9.455	3.278-27.275	<.001	8.475	2.871-25.011	<.001

In both the analyses, all cases or early breast cancer alone, high 53 expression did not result an independent prognostic factor in the multivariate analysis.

## Discussion

*TP53* mutations represent the most frequent genetic abnormality in human cancer. They are observed in 20%-30% of all primary invasive breast cancer and missense mutations account for up to 90% of the cases. Missense mutations generally lead to a dysfunctional mutant p53 protein with an increased stability that tends to accumulate in the nucleus. Therefore, IHC determination of p53 expression has been proposed and used as a surrogate for TP53 status. In the series presented by Schmitt et al and Alsner et al, there was a strong relation between p53 accumulation and TP53 mutations, while Norberg et al reported a sensibility and specificity of 72.2% and 92%, respectively for IHC compared to DNA sequencing. Various authors have provided different cutoffs to define p53 overexpression, but to date no suitable value has been established. The most frequently reported cutoff values are set at either 10% (Millar et al) or 50% (Kikuchi et al). In our cohort, 50% was deemed a reasonable cutoff, as it correlated more strongly to outcome. Null mutations (nonsense and frameshift) are less common and lead to a truncated nonfunctional mutant p53 protein. They are associated with a total absence of immunostaining for p53.^12,29^ The biological differences and prognostic impact between missense and null mutations are still not clear. Various authors have investigated the role of different types of *TP53* mutations in human cancer, but the results remain controversial.^13,30-33^ In the present study, we consider the surrogates of missense and null mutations separately and propose a new classification based on p53 IHC expression: null (0%), low (0.1%-49%) and high (≥50%). Such classification has not been previously reported in literature to our best knowledge.

The prognostic impact of p53 status in breast cancer is still not fully clarified. Wild-type *TP53* has been correlated to worse outcome in patients receiving chemotherapy, while mutant-p53 has been linked to a negative prognostic impact in patients treated with endocrine therapy alone.^14^ The role of p53 expression determined by IHC is still controversial. Several authors have reported an association between p53 overexpression and worse outcome in patients with luminal-like breast cancer. Lee et al found such association only among luminal A-like tumors, while in the series presented by Reed et al no significant correlation between p53 accumulation and survival was observed. In our cohort, high p53 expression was associated with shorter DFS and DRFS only in the univariate analysis. On the contrary, null p53 resulted a significant and independent prognostic factor for both DFS and DRFS. Null p53 is more likely to be observed in case of nonsense or frameshift TP53 mutations for which a negative prognostic impact has already been reported. These findings were confirmed also in the analysis of patients with early breast cancer. Due to lack of complete data, we could not analyze the differences among groups based on the type of postsurgical therapy received.

As our findings suggest, patients with null p53 are at higher risk, thus systemic adjuvant therapy might prove beneficial in terms of disease-free survival in these cases, particularly among patients with early breast cancer. As a monocentric study, the strongest point of our work is the homogeneity of the sample, loco-regional treatment, and pathological evaluation, particularly in an era with fewer therapeutic options and when tumor stage played a primary role in treatment decisions rather than the molecular profile. On the other hand, it has the limits of a retrospective study and a limited sample of cases and data on postoperative therapy. Further studies are required to investigate the role of p53 status on survival distinguishing on the type of postsurgical treatment received.

## Conclusion

Integrating p53 status determined by IHC with the traditional clinicopathological features in patients with HR-positive/Her2-negative breast tumors can be a valuable tool in the hands of the clinician in the decision of the most appropriate therapy, particularly in patients with early breast cancer and null p53 expression.

## Data Availability

The data underlying this article are available in the article and in its online supplementary material
